# Polysaccharide enhances Radix Saposhnikoviae efficacy through inhibiting chromones decomposition in intestinal tract

**DOI:** 10.1038/srep32698

**Published:** 2016-09-06

**Authors:** Jing-Ming Yang, Hua Jiang, Hong-Liang Dai, Zi-Wei Wang, Gui-Zhi Jia, Xiang-Cai Meng

**Affiliations:** 1 College of Pharmacy, Heilongjiang University of Chinese Medicine, Harbin 150040, China; 2 College of Pharmacy, Jinzhou Medical University, Jinzhou 121000, China; 3 School of Nursing, Jinzhou Medical University, Jinzhou 121000, China; 4 Department of Biochemistry and Molecular Biology, Jinzhou Medical University, Jinzhou 121000, China

## Abstract

Vegetative but not reproductive stage of *Saposhnikovia divaricate* (Turxz.) schischk possesses pharmacological activities. However, our recent study showed that reproductive *S. divaricate* supplemented with polysaccharide showed evidently elevated pharmacological activities and increased cimifugin content in rat serum. The aims of present study were to assess the influence of polysaccharides on the chromones pharmacological activities in Radix Saposhnikoviae (RS), the dried root of vegetative stage of *S. divaricate*, and to explore the underlying mechanisms. Only cimifugin was detected in the plasma of chromone treated animals and RS polysaccharide significantly increased the plasma content of cimifugin. It was shown that neither cimifugin absorption nor glycoside components transformation in simulated digestive fluid was affected by RS polysaccharide. However, a significant promotion of transformation of cimifugin to more stable prime-O-glucosylcimifugin (PGCN) by RS polysaccharide, and a protective effect of polysaccharide on chromone components were observed in small intestine solutions. Meanwhile, RS polysaccharide produced a significant elevation of cimifugin and PGCN concentration *in vivo*. Based on these findings, we concluded that RS polysaccharide could greatly increase the content of cimifugin, which might be related to its degradation-proof effect on cimifugin, via transforming cimifugin to comparatively more stable PGCN and spatial structure protection.

Radix Saposhnikoviae (RS) is the dried root of vegetative stage of perennial herb *Saposhnikovia divaricate* (Turxz.) schischk in family Umbelliferae, and is commonly used for treating febrility, rheumatism, headache, vertigo, generalized aching, convulsion, arthralgia and inflammatory symptoms for thousands of years in China, Japan, and Korea^1^,^2^,^3^,^4^,^5^. Modern scientific experiments have also confirmed the beneficial effects of RS using its extracts^1^,^5^,^6^,^7^. RS contains various bioactive substances, such as chromones, coumarins, polyacetylenes, and polysaccharides. Among them, chromones and coumarins are the main components of RS^4^,^8^,^9^. Furthermore, chromones have also been identified as the main bioactive constituents with most-evident pharmacological activities as they possess potent analgesic, antifebric, anti-inflammatory and immune-regulatory effects^1^,^5^.

Currently, three major types of chromones were found in RS, including prime-O-glucosylcimifugin (PGCN), 4′-O-β-D-glucosyl-5-O-methylvisamminol (GML), and cimifugin (Fig. 1). In Chinese pharmacopoeia, PGCN and GML are designated as marker compounds for RS quality control due to their relatively high content and GML is also used for the identification of RS in Japanese Pharmacopoeia^3^,^4^,^10^,^11^. Cimifugin is aglycone of PGCN and has much lower content than that of PGCN and GML. Huge amount of cimifugin and trace amount of PGCN and GML were detected in the plasma sample of overdose chromone^4^,^12^. Generally, cimifugin is regarded as the pharmacologically active form of inactive chromone derivatives such as PGCN and GML, which are transformed into bioavailable cimifugin in the gastrointestinal tract^4^,^7^,^11^,^12^.

In addition to chromones, several other types of components were also identified in RS, including polysaccharides, coumarins, volatile oils, polyacetylenes, organic acids and so on^4^,^8^,^9^. Among these constituents, polysaccharides represent as immune-regulatory and antineoplastic components in RS^13^,^14^. Apart from its direct pharmacological activities, mounting researches showed that polysaccharides might indirectly effected on other active ingredients in medicinal herbs via influencing their pharmacokinetic process. For example, previous report showed that gastrodin, an effective constituent in Chinese traditional medical herb *Rhizoma gastrodiae*, exhibited greatly decreased retention time and serum content when polysaccharides were removed from this plant^15^. A significant increase in absorption and decrease in elimination of the effective components in *Schisandra chinensis* was also observed when this herb was administered in combination with polysaccharide-rich *Radix ophiopogonis*^16^. In our present study, we are focusing on RS (traditionally the officinal part is the dried root of its vegetative stage). Once entering the stage of reproductive growth, its medicinal value becomes nil. Intriguingly, relevant research revealed that it was the content of polysaccharide rather than active chromone substances that greatly decreased in reproductive stage of RS^17^. Furthermore, our recent study showed that when supplemented with polysaccharides, reproductive stage of RS showed enhanced antipyretic, analgesic, anti-inflammatory activities with evidently elevated content of cimifugin in rat serum^18^. Based on these facts, we reckoned that polysaccharide component might play an essential role when RS exerting its pharmacological activity, perhaps via improving the pharmacokinetic process of chromone ingredients. Therefore, the aims of present study were to determine the beneficial effect of RS polysaccharides on chromone pharmacokinetics and to clarify the underlying mechanisms.[Fig f1][Fig f2]

## Materials and Methods

### Reagents

Cimifugin, prime-O-glucosylcimifugin (PGCN), and 4′-O-β-D-glucosyl-5-O-methylvisamminol (GML), with all ≥ 98% purity, were provided by Shanghai Jinsui Bio-Technology Co., Ltd (Shanghai, China). LY335979 (Zosuquidar Trihydrochloride) was provided by Shanghai Mingrong Bio Co., Ltd (Shanghai, China). Troleandomycin was bought from Hubei Jusheng Technology Co. Ltd. Glucose was obtained from Shanghai Jingke Chemical Technology Co., Ltd (Shanghai, China). Glucuronic acid was obtained from Shenyang Jiuye Chemical Material Co., Ltd (Shenyang, China). HPLC-grade methanol was purchased from Tianjin Guangfu Fine Chemical Research Institute (Tianjin, China). Pepsin was obtained from Sigma-Aldrich (St. Louis, MO, USA). Other chemicals and solvents were analytical purity grade. Distilled water prepared from demineralized water was used throughout the experiment.

### Animals

Animal experiments were conducted in accordance with the guidelines of the National Institutes of Health (NIH guidelines) and approved by the Ethical Committee of Heilongjiang University of Chinese Medicine (approval number: HUCM2014-00348). Sprague-Dawley rats weighing 180~220 g were maintained under a 12/12h light/dark cycle and kept at controlled temperature (25 ± 1 °C) and humidity (70 ± 10%) conditions. The rats were provided free access to standard laboratory diet and water. All efforts were made to guarantee minimally used animal number and suffering. Not any adverse health effects on animals were found with the test compounds.

### Sample preparation

RS, the roots of *S.divaricata* (Turcz) schischk, were bought from Beijing Tong Ren Tang Group Co. Ltd and identified by Professor Xiang-Cai Meng, College of Pharmacy, Heilongjiang University of Chinese Medicine. The chopped dried roots were extracted thrice with distilled water at the reflux temperature and the solutions were concentrated into viscous state. Subsequently, about seven fold of 95% ethanol was added, this ethanol solution was set aside overnight and then subjected to decompress filtration followed by separate collection of filtrated liquid and filter residue. The filtrated liquid, which were just the chromone components in RS, were divided into four equal aliquots for follow-up experiments. Then the filtrate residues were washed with 95% ethanol, acetone and petroleum ether successively and underwent deproteinization and decoloration. The decolorated residues were eluted with distilled water with the effluent collected when Molish reaction was positive. This collection was stopped when Molish reaction exhibited negative. After decompressed filtration and freeze-drying, a total of 8.44 g RS polysaccharide was obtained.

### Pharmacokinetic measurement of chromone

To explore the influence of RS polysaccharide on the pharmacokinetics of chromones, 2.11 g, 1.05 g RS polysaccharide, and 2.11 g glucuronic acid were added to the three aliquots of chromone components, with one aliquot left as control. Thus, totally four test compounds were obtained, including chromone/pan-polysaccharide (CP), chromone/1/2polysaccharide (CHP), chromone/glucuronic acid (CG), and chromone, which served as control. Forty male Sprague-Dawley rats were used in this experiment. They were randomly divided into 4 groups, each consisting of 10 rats. The rats in these four groups were orally administered with CP, CHP, CG and chromones at the dose of 4 g crude drug (equivalent to 10 ml RS extracts) per kg body weight, respectively for five consecutive days. After the final drug delivery of day 5 (at 0.5, 1, 1.5, 2, 3, 5, 8, 12, 16, and 24 h/day), rats were anesthetized by ether inhalation and rat blood samples were obtained from the orbit vein and centrifuged immediately at 3,000 rpm for 10 min to yield plasma. Each plasma sample (100 μl) was mixed with 20 μl of 70% perchloric acid and vortexed and centrifuged at 3,000 rpm for 10 min. The supernatant were subjected to 0.45 μm microporous membrane and the filtrate was collected for subsequent high performance liquid chromatography (HPLC) analysis. Chromatographic separation and analysis using an L-2000 Elite HPLC system has been previously reported^7^. Briefly, the separation of the analytes were performed on a Kromasil C_18_ column (200 mm × 4.6 mm i.d., particle size 5 μm), equipped with a Shim-pack security guard column at a column temperature of 25 °C. The binary mobile phase consisted of methanol and water. A flow rate of 1.0 ml/min with a gradient elution program as follows: 40–45% methanol at 0–5 min; 45–60% methanol at 5–10 min; 60–80% methanol at 10–15 min; 80–95% methanol at 15–20 min; 95–40% methanol at 20–30 min. The injection volume was 20 μl and detection wavelength was kept at 254 nm.

### Caco-2 cell culture and bidirectional transport assay

Caco-2 cells were obtained from the American Type Culture Collection (ATCC). Cells were maintained in high glucose DMEM media supplemented with L-glutamine, 10% fetal bovine serum, 1% nonessential amino acids and 1% penicillin/streptomycin in a humidified atmosphere of 5% CO_2_ at 37 °C. For the permeability test, cells were seeded in transwell supports with polycarbonate membrane (0.4 μm, 1.12 cm^2^, Corning Costar) at a density of 6 × 10^4^ cells/cm^2^. The integrity of the monolayers was tested by measuring the transepithelial electrical resistance (TEER) before and after every experiment. Only monolayers with TEER >200 Ω/cm^2^ were used. Test compounds in transport buffer were added to the donor compartments of either apical (A → B) or basolateral (B → A) wells of the Insert System and incubated at 37 °C. Samples were collected at 0, 30, 60, 90, 120, and 150 min time points and analyzed for HPLC analysis of content of cimifugin as described above.

### Metabolism studies of PGCN and GML in SGF and SIF

Simulated gastric fluid (SGF, pH 1.2) and simulated intestinal fluid (SIF, pH 6.8) were prepared based on Chinese Pharmacopeia (2010) and have been depicted previously^7^,^9^. 100 μg PGCN or GML was added to 4 ml of either SGF or SIF to yield 4 reaction systems, i.e., PGCN/SGF (213.47 nM), PGCN/SIF (213.47 nM), GML/SGF (220.99 nM), GML/SIF (220.99 nM), respectively. Polysaccharides extracted in RS were added to these 4 reaction systems in accordance with the actual chromone-polysaccharide proportion, with each reaction further became three sub-systems, i.e., 0 polysaccharide/chrome, 1/2 polysaccharide/chromone, and pan-polysaccharide/chromone. Meanwhile, these 12 systems were allowed to react at 37 °C. Following 2 h, the reaction was stopped by 100 °C boiling for 10 min. Subsequently, these systems were evaporated to dryness and the residue was reconstituted in 1 ml methanol. After centrifugation at 3,000 rpm for 10 min, the supernatant was collected and used for HPLC analysis.

### Preparation of enzyme solution from small intestine and grouping

Small intestine segment was taken from ten 12 h-fasting male Sprague-Dawley rats. The intestine tissue was first frozen at −80 °C for 12 h, and then ground and centrifuged at 10,000 rpm for 10 min. The supernatant constituted the enzyme solution used in the experiment. To explore the influence of RS polysaccharide on the metabolism of cimifugin, three groups were designed: control group containing 40 μg cimifugin in 1.2 ml enzyme solution; RS polysaccharide/cimifugin group containing 40 μg cimifugin and 8.7 mg RS polysaccharide in 1.2 ml enzyme solution; glucuronic acid/cimifugin group containing 40 μg cimifugin and 8.7 mg glucuronic acid in 1.2 ml enzyme solution. These groups were put in 38 °C water bath for reaction, while samples were taken at 15 and 30 min. After deproteinization by methanol, these samples were centrifuged at 10,000 rpm for 10 min. Subsequently, the supernatant was collected and analyzed for HPLC analysis as described above.

### Transport study of chromones across semi-permeable membrane in the absence or presence of RS polysaccharide

Dialysis bags made by semi-permeable membrane materials were used in this analysis. ~3 cm long dialysis bags were first processed as the following procedures in succession: 1) boiled for 5 min in distilled water; 2) rinsed by 60 °C water for 2 min; 3) rinsed by small intestine buffer for 2 min; 4) rinsed by small intestine buffer for 2 min; 5) immersed in buffer at 4 °C. After 12 h, these processed dialysis bags were tied tightly at one end to form a dialysis bag. 1 ml small intestine buffer (pH 7.8) containing cimifugin, PGCN, cimifugin + polysaccharide, or PGCN + polysaccharide was added into this bag and a glass bead was put in to keep this bag vertical. When the other end was tied (an air bubble was retained), this dialysis bag was suspended in 15 ml small intestine buffer. This system was left to stand for 6 h in 37 °C water bath and chromone content inside and outside of this semi-permeable membrane was measured by HPLC analysis.

## Results

### Pharmacokinetic study of chromone in rat plasma

**Figure 2 f2:**
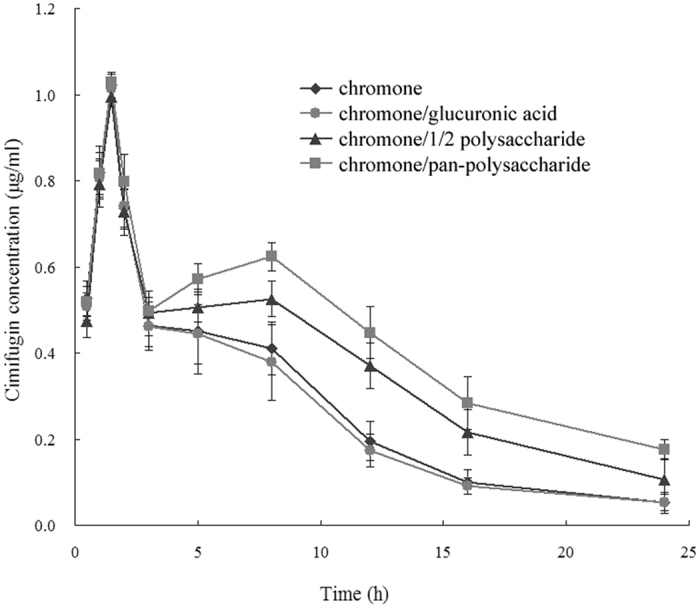
The concentration-time curve of cimifugin in rat plasma after oral administration of chromone extracts in the presence of glucuronic acid or Radix Saposhnikoviae (RS) polysaccharides. The rats were orally administered with chromone, chromone/glucuronic acid (CG), chromone/1/2 polysaccharie (CHP), and chromone/pan-polysaccharide (CP), respectively. Blood samples from rats were then collected 0.5, 1, 1.5, 2, 3, 5, 8, 12, 16, and 24 h following the drug delivery for high performance liquid chromatography (HPLC) analysis.

In this study, HPLC qualitative analysis showed that only cimifugin was detected in the plasma of chromone treated rats in the whole process of the experiment (data not shown). Based on the qualitative results, cimifugin concentrations in rat plasma were further quantitatively measured. As seen in Fig. 2, the concentration-time relationship exhibited as a double-peak curve, with T_max_ at 1.5 and 8 h, respectively. In the first three hours, not any difference of AUC value was found among the four groups as indicated in the figure, however, a significant distinction of AUC was seen among them at 3 h and later. Specifically, glucuronic acid did not affect the absorption process of chromone, but polysaccharide could significantly increase the absorption of chromone in the form of cimifugin. Moreover, pan-polysaccharide displayed comparatively greater effect than 1/2 polysaccharide.

### Influence of RS polysaccharide on transport of cimifugin after apical or basolateral application

As seen in Table 1, a time-dependent flux of cimifugin from the apical-to-basolateral (A → B) direction was observed after apical application of cimifugin. This flux was significantly enhanced by pretreatment of troleandomycin, a specific inhibitor of CYP3A, but not affected by LY335979, a specific inhibitor of P-glycoprotein inhibitor. Similar results was observed when cimifugin was applied in basolateral chamber (Table 2, B → A transport), thereby suggesting a CYP3A4 activity-dependent process of cimifugin transport. RS polysaccharide produced no effect on cimifugin transport either in the presence or absence of inhibitors. As such, it was concluded that RS polysaccharide was unable to affect the flux across the intestine tract.

### Metabolism studies of PGCN and GML in SGF and SIF

The metabolic profiles of PGCN and GML in the absence and presence of RS polysaccharide in simulated gastrointestinal fluid were investigated. In SGF, a small amount of PCGN was converted into cimifugin, and a little GML was converted into PGCN (Table 3). In SIF, an overwhelmingly large amount of PGCN was transformed into cimifugin, and GML underwent no change (Table 4). Neither in SGF (Table 3) nor in SIF (Table 4), there was significant influence of polysaccharide on the transformation of these two glycosides in RS.

### Effect of RS polysaccharide on the transformation of cimifugin in small intestine enzyme solutions

As shown in Table 5, cimifugin underwent degradation to a great extent in small intestine enzyme solutions, with small amount of which transformed into PGCN. Intriguingly, although RS polysaccharide produced no effect on cimifugin degradation, it promoted quite amount of cimifugin transformed into PGCN. After removing or denaturing the small intestine enzymes, RS polysaccharide still evoked this transformation to a similar extent. In contrast, as shown in Table 6, glucuronic acid and glucose did not produce any effect on cimifugin transformation. All these findings indicated that RS polysaccharide promoted the transformation of cimifugin to PGCN in a direct manner, rather than via affecting the bioactivity of the small intestine enzymes.

### Effect of RS polysaccharide on the trans-membrane transport of chromones

As shown in Table 7, RS polysaccharide produced an 81.12% elevation of cimifugin concentration inside the semi-permeable membrane. Likewise, RS polysaccharide also caused a 32.24% elevation of PGCN concentration inside the semi-permeable membrane. These findings indicated that polysaccharide might have a remarkable “protective” effect on chromone components in intestine tracts.

## Discussion

PGCN, GML and cimifugin are the three major chromones in RS with clear chemical structures. Although the content of cimifugin is the lowest among these three components in RS, it represented as the main absorbed and active component in plasma after oral administration of RS extract. In contrast, the other two chromones possess feeble pharmacological activities and are hard to be detected by HPLC when used at regular doses. It was generally speculated that these two glycosides might function after firstly biotransformed into cimifugin in the gastrointestinal tract, then the latter was absorbed into the blood^4^,^11^. As such, we used cimifugin here as the indicative component. Our present study showed that RS polysaccharide significantly promoted the absorption of cimifugin, with an obvious dose-response relationship. In the presence of polysaccharide, AUC_0–24_ of cimifugin showed a 0.56-fold increase, compared with sole administration of chromones. The polysaccharide structure might be essential for this absorption promoting effect, as this effect was not found with monosaccharides, such as glucose.

Subsequently, the underlying mechanism of RS polysaccharide increasing cimifugin content in plasma was also explored. CYP3A4 accelerates the decomposition of exogenous foreign bodies, hindering their absorption while, P-glycoprotein promotes the exocytosis of exogenous foreign bodies. Both of these proteins are the key to limit the absorption of exogenous agents through gastrointestinal tract. In order to figure out whether CYP3A4 and P-glycoprotein were involved in the cimifugin absorption promoting process of RS polysaccharide, two pharmacological inhibitors, LY33597 and troleandomycin targeting CYP3A4 and P-glycoprotein, were introduced, respectively. Our results revealed that LY353597 failed to increase the absorption of cimifugin, suggesting that cimifugin was not likely to be destroyed by intestinal CYP3A4. In contrast, troleandomycin has a significant influence on cimifugin content, indicative of an exocytosis promoting effect of P-glycoprotein. Besides, polysaccharide did not affect the transport of cimifugin between side A and B, and not increase the inhibitory effect of the two above-mentioned pharmacological inhibitors. Based on these, it was concluded that it was not through inhibiting P-glycoprotein and CYP3A that polysaccharide promoted the absorption of cimifugin.

PGCN is transformed into cimifugin by enzymes in gastrointestinal fluid. RS extract contained a huge amount of PGCN and traces of cimifugin. This had a significant bearing on the large quantities of transformation of PGCN into cimifugin at the very beginning. PGCN did not easily transport across the cell membrane for absorption due to its high polarity, whereas cimifugin was readily absorbed into blood^4^. The production of cimifugin was dominated at the beginning, when the amount of absorbed cimifugin was dependent on its generation. As such, it is understandable that a similar level of plasma content of cimifugin was found among groups within the first three hours. There are two active –OH in cimifugin molecule, which leads to its rapid degradation before absorption: the higher the cimifugin concentration, the more amount of degradation of this substance. Considerable breakdown of cimifugin in intestinal tract would result in the decrease of cimifugin in the blood. The amount of PGCN in intestinal tract was gradually decreased, when the reaction of cimifugin proceeded towards PGCN. On the other hand, the content of cimifugin steeply enhanced. As such, polysaccharide preventing cimfugin from degrading matters.

Above all, polysaccharide can convert cimifugin into chemically more stable PGCN. In view of the facts that: 1) the glycoside group of PGCN and GML is of glucose; 2) glucuronyl transferase exists in the small intestine of human and rats and is essential for glycoside synthesis, we initially reckoned that the polysaccharide might exert its protective effect depending on glucose and glucuronyl transferase. However, our results did not support this hypothesis, as these substances failed to increase the production of PGCN and GML. That is to say, polysaccharide itself was the main material basis for cimifugin transformation into PGCN. This experiment exploring the effect of polysaccharide on cimifugin transformation revealed that the relatively lower content of cimifugin was due to the presence of the transformation process from cimifugin to PGCN. RS polysaccharides are of macromolecular active substance with complex spatial structure and active groups, such as -OH and -COOH. It is still not clear how cimifugin and polysaccharide react. It is speculated that structurally complex polysaccharide catalyze the transformation from cimifugin to more stable PGCN and GML, thereby averting its breakdown. In addition, the complex spatial structure of polysaccharide might also provide physical protection to cimifugin and PGCN^19^. Cimifugin and PGCN might bind to polysaccharide through a certain manner after entry into this complex spatial structure, thus protecting them against being destroyed by gastrointestinal enzymes.

Transformation from PGCN to cimifugin increases the absorption of chromone, whereas the opposite process decreases the breakdown of chromones before their absorption. Polysaccharide was able to prevent chromones from being destroyed in intestinal tract. There exists a dynamic balance of the inter-transformation between RS chromone glycosides and its aglycone. The protective effect of polysaccharide on chromone transformation would increase the therapeutic effect of chromones. The present finding afforded us salutary lessons with regard to the quality control and clinical application of RS and other medicinal materials.

## Additional Information

**How to cite this article**: Yang, J.-M. *et al.* Polysaccharide enhances Radix Saposhnikoviae efficacy through inhibiting chromones decomposition in intestinal tract. *Sci. Rep.* **6**, 32698; doi: 10.1038/srep32698 (2016).

## Figures and Tables

**Figure 1 f1:**
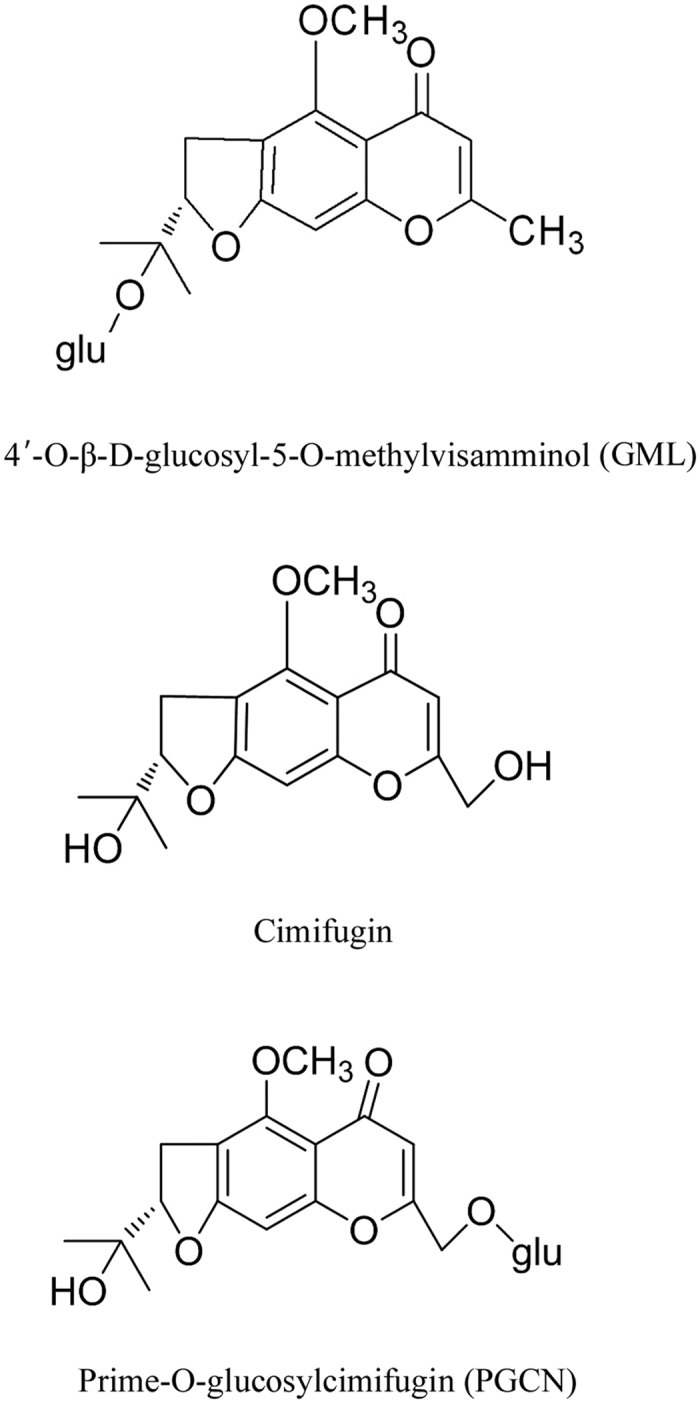
Chemical structures of 4′-O-β-D-glucosyl-5-O-methylvisamminol (GML), cimifugin, and prime-O-glucosylcimifugin (PGCN)

**Table 1:** Time-dependent transport of cimifugin in Caco-2 cell monolayers after apical application of different test compounds.

Test Compound	Basolateral content of cimifugin (ng)				
	30 min	60 min	90 min	120 min	150 min
A	15.21 ± 2.34	30.85 ± 3.64	143.92 ± 12.32	236.01 ± 22.86	201.33 ± 15.32
A/B	13.53 ± 4.22	31.35 ± 4.74	137.11 ± 9.46	222.66 ± 18.43	212.51 ± 20.47
A/C	16.32 ± 1.97	31.37 ± 4.64	142.82 ± 11.63	231.42 ± 24.55	204.35 ± 24.52
A/B/C	16.53 ± 2.01	32.67 ± 4.4	144.76 ± 12.23	237.12 ± 21.85	200.43 ± 10.11
A/D	48.54 ± 3.57*	110.59 ± 11.50*	247.57 ± 17.00*	316.65 ± 31.53*	252.83 ± 20.35*
A/B/D	46.36 ± 6.46*	104.63 ± 14.89*	258.54 ± 18.58*	325.34 ± 25.61*	249.25 ± 17.54*

A: cimifugin; B: polysaccharide; C: LY335979; D: troleandomycin.

Data were presented as mean ± SD (n = 10). ^*^P < 0.05 versus A group.

**Table 2:** Time-dependent transport of cimifugin in Caco-2 cell monolayers after basolateral application of different test compounds.

Test compounds	Apical content of cimifugin (ng)				
	30 min	60 min	90 min	120 min	150 min
A	26.87 ± 1.54	50.45 ± 2.27	234.35 ± 11.36	354.24 ± 15.47	289.46 ± 15.45
A/B	23.42 ± 1.65	52.23 ± 3.62	246.53 ± 13.34	348.84 ± 21.34	294.33 ± 13.67
A/C	25.25 ± 2.23	52.32 ± 4.54	263.33 ± 20.42	344.44 ± 21.34	278.35 ± 23.34
A/B/C	24.35 ± 1.87	49.24 ± 3.64	246.53 ± 18.32	351.26 ± 20.11	282.42 ± 21.18
A/D	20.85 ± 1.68*	44.24 ± 3.24*	225.44 ± 17.66*	332.64 ± 22.85*	256.66 ± 18.47*
A/B/D	21.99 ± 2.26*	46.82 ± 3.26*	233.85 ± 16.90*	326.5 ± 16.87*	253.65 ± 20.79*

A: cimifugin; B: polysaccharide; C: LY335979; D: troleandomycin.

Data were presented as mean ± SD (n = 10). ^*^P < 0.05 versus A group at the same time point.

**Table 3:** Metabolic study of PGCN and GML in SGF in the absence and presence of RS polysaccharide.

Test compounds	HPLC peak area		
	Cimifugin	PGCN	GML
PGCN	33145 ± 2233	966390 ± 35555	N.A.
PGCN/1/2 Polysaccharide	26204 ± 1425	931227 ± 23420	N.A.
PGCN/Pan-polysaccharide	36062 ± 2151	936065 ± 42384	N.A.
GML	N.A.	22814 ± 4238	1369608 ± 83550
GML/1/2 Polysaccharide	N.A.	23657 ± 2427	1389523 ± 93428
GML/Pan-polysaccharide	N.A.	22253 ± 2154	1218659 ± 42338

Data were presented as mean ± SD (n = 10).

**Table 4:** Metabolic study of PGCN and GML in SIF in the absence and presence of RS polysaccharide.

Test compounds	HPLC peak area		
	Cimifugin	PGCN	GML
PGCN	1101759 ± 81002	5159 ± 1238	N.A.
PGCN/1/2 Polysaccharide	1131570 ± 103451	5698 ± 2214	N.A.
PGCN/Pan-polysaccharide	1291256 ± 42563	5488 ± 2137	N.A.
GML	N.A.	N.A.	1369720 ± 112480
GML/1/2 Polysaccharide	N.A.	N.A.	1303171 ± 98550
GML/Pan-polysaccharide	N.A.	N.A.	1651263 ± 132537

Data were presented as mean ± SD (n = 10).

**Table 5:** Transformational study of cimifugin to PGCN and GML in the absence and presence of RS polysaccharide.

Reaction time	Test compounds	Chromone content (μg/mL)	PGCN	GML
		Cimifugin		
15 min	A/B	1.52 ± 0.16	39.88 ± 2.57	0.00 ± 0.00
	A/B/C	13.65 ± 1.11*	38.27 ± 2.45	1.09 ± 0.12*
	A/C	16.58 ± 1.26*	65.74 ± 3.68*	2.74 ± 0.31*
	A/C/D	16.92 ± 1.06*	68.60 ± 4.62*	2.92 ± 0.50*
30 min	A/B	2.82 ± 0.14	32.26 ± 2.01	0.00 ± 0.00
	A/B/C	13.79 ± 0.99*	34.13 ± 2.46	1.31 ± 0.12*
	A/C	20.71 ± 1.88*	56.36 ± 3.56*	2.95 ± 0.14*
	A/C/D	22.68 ± 1.66*	58.43 ± 5.37*	2.94 ± 0.52*

A: cimifugin; B: enzyme; C: polysaccharide; D: denatured enzyme.

Data were presented as mean ± SD (n = 10). ^*^P < 0.05 vs “A/B” group at the same time point.

**Table 6:** Transformational study of cimifugin to PGCN and GML in the absence and presence of glucuronic acid and glucose.

Reaction time	Chromone content (μg/mL)			
	Test compounds	Cimifugin	PGCN	GML
15 min	A/B	1.52 ± 0.16	39.88 ± 2.57	0.00 ± 0.00
	A/B/C	4.35 ± 0.32	38.25 ± 2.23	0.00 ± 0.00
	A/B/D	6.70 ± 0.67	21.95 ± 2.01	0.00 ± 0.00
30 min	A/B	2.82 ± 0.14	32.26 ± 2.01	0.00 ± 0.00
	A/B/C	4.73 ± 0.41	33.66 ± 2.58	0.00 ± 0.00
	A/B/D	8.89 ± 1.05	22.88 ± 1.40	0.00 ± 0.00

A: cimifugin; B: enzyme; C: glucuronic acid; D: glucose.

Data were presented as mean ± SD (n = 10).

**Table 7:** Protective effect of polysaccharide on cimifugin and PGCN.

Groups	Fluid outside the semi-permeable membrane			Fluid inside the semi-permeable membrane			total chromone (nmol)
	PGCN (nmol)	cimifugin (nmol)	chromone (nmol)	PGCN (nmol)	cimifugin (nmol)	chromone (nmol)	
Cimifugin	N.A.	143.6 ± 0.1	143.6 ± 0.1	N.A.	18.1 ± 0.6	18.1 ± 0.6	161.7 ± 0.5
Cimifugin/polysaccharide	30.3 ± 1.5**	98.1 ± 0.3**	128.4 ± 1.4*	6.3 ± 0.1**	26.4 ± 1.2	32.7 ± 1.2**	161.1 ± 0.4
PGCN	89.8 ± 0.4	N.A.	89.8 ± 0.4	15.2 ± 0.5	N.A.	15.2 ± 0.5	105.0 ± 0.1
PGCN/polysaccharide	85.7 ± 0.6#	N.A.	85.7 ± 0.6#	20.1 ± 0.2**	N.A.	20.1 ± 0.2##	105.8 ± 0.7

Data were presented as mean ± SD (n = 10). ^*^P < 0.05, ^**^P < 0.01 vs. “cimifugin” group; ^#^*P* < 0.05, ^##^*P* < 0.01 vs. “PGCN” group.
